# Co-infection with HPV types from the same species provides natural cross-protection from progression to cervical cancer

**DOI:** 10.1186/1750-9378-9-26

**Published:** 2014-08-12

**Authors:** Rafal S Sobota, Doreen Ramogola-Masire, Scott M Williams, Nicola M Zetola

**Affiliations:** 1 Center for Human Genetics Research, Department of Molecular Physiology and Biophysics, Vanderbilt University Medical Center, Nashville, TN, USA; 2 Department of Genetics, Geisel School of Medicine, Dartmouth College, Hanover, NH, USA; 3 Division of Infectious Disease, University of Pennsylvania, Philadelphia, PA, USA; 4 Botswana-University of Pennsylvania Partnership, 214 Independence Ave, Gaborone, Botswana; 5 Department of Medicine, University of Botswana, Gaborone, Botswana; 6 Center for Human Genetics Research, Vanderbilt University, 519 Light Hall, 2215 Garland AveNashville, TN 37232, USA

**Keywords:** HPV, Taxonomy, Cervical cancer, Coinfection, Immune cross-protection, Vaccine

## Abstract

**Background:**

The worldwide administration of bivalent and quadrivalent HPV vaccines has resulted in cross-protection against non-vaccine HPV types. Infection with multiple HPV types may offer similar cross-protection in the natural setting. We hypothesized that infections with two or more HPV types from the same species, and independently, infections with two or more HPV types from different species, associate with protection from high-grade lesions.

**Findings:**

We recruited a cohort of 94 HIV, HPV-positive women from Botswana, with Grade 2 or higher cervical intraepithelial neoplasia. Infections with 2 or more HPV types from a single species associated with reduced lesion severity in univariate analysis (OR = 0.41, 95% CI 0.18-0.97, p = 0.042), when adjusted for the presence of HPV 16 or 18 types (OR = 0.41, 95% CI 0.17-1.00, p = 0.049), or all high-risk HPV type infections (OR = 0.38, 95% CI 0.16-0.90, p = 0.028). Infections with 2 or more HPV types from different species did not associate (OR = 0.68, 95% CI 0.25-1.81, p = 0.435).

**Conclusions:**

Our findings show that co-infections with genetically similar HPV types reduce the likelihood of progression to high-grade lesions in HIV positive women, an effect not observed in co-infections with taxonomically different HPV types. This observation is possibly caused by an immune cross-protection through a similar mechanism to that observed after HPV vaccination.

## Background

Cervical cancer is the third most common cancer in women worldwide and the number one cause of cancer-related mortality in Sub-Saharan Africa [1]. Infection with an oncogenic human papillomavirus (HPV) type is necessary, but not sufficient, for progression to cervical cancer [2]. Most HPV types do not cause cancer, and position on the viral phylogeny affects disease risk [3,4]. HPV phylogeny is based on genetic distance between isolates, with the genus serving as the broadest taxon. Within a genus, similar viruses are classified into species, and viruses within a species are subdivided into types [5,6]. Only the alpha genus of HPV is associated with cervical cancer [7]. The carcinogenic HPV types fall into 5 species, α5, α6, α7, α9 and α11 [3] and are termed high-risk species. Two HPV types, 16 and 18, are responsible for approximately 70% of cervical cancer cases worldwide; with the majority of the remainder ascribed to 11 other types [8].

Vaccination against HPV was a major advance in cervical cancer prevention, offering protection against a few disease causing types. Currently, 2 prophylactic vaccines are approved for human use. The tetravalent vaccine covers four HPV types, offering protection against cervical cancer (types 16 and 18) as well as genital warts (types 6 and 11). The bivalent vaccine covers types 16 and 18, targeting only cervical cancer [5,6,9]. Understanding the effects of HPV co-infections is essential in determining the long term effects of the vaccines. Availability of such results can better inform the projections on the effect of broad vaccine implementation, and the subsequent prevalence of cervical cancer. Available data suggest that both vaccines have a variable level of cross-protection against types closely related HPV types [10]. It is hypothesized that this protection can be attributed to the genetic similarity between the vaccine types and those against which cross-protection is afforded, as the types for which cross-protection was observed are in the same species as HPV 16 and 18.

In this study we determined whether taxonomic relationships of co-infecting HPV types associated with progression to advanced cervical lesions (CIN3+). We tested two independent hypotheses: 1) infections with two or more HPV types from the same species associate with progression to high-grade lesions; or 2) infections with two or more HPV types from different species associate with progression to high-grade lesions.

## Findings

We recruited HIV-positive women living in Botswana who were diagnosed with a Grade 2 or higher cervical intraepithelial neoplasia (CIN2 or above) on colposcopy. All patients provided informed consent.

Specimen collection and processing have been previously described [11]. Briefly, Pap smear swabs were lysed in 500 μL of lysis buffer (Roche MagNA Pure LC DNA Isolation Kit) for 30 minutes at room temperature. DNA was extracted, amplified and analyzed following the manufacturer’s specifications (Roche Linear Array® HPV Genotyping test). This method can detect 13 high-risk HPV genotypes (genotypes 16, 18, 31, 33, 35, 39, 45, 51, 52, 56, 58, 59, and 68) and 24 low-risk HPV genotypes (genotypes 6, 11, 26, 40, 42, 53, 54, 55, 61, 62, 64, 66, 67, 69, 70, 71, 72, 73, 81, 82, 83, 84, IS39, and CP6108).

HPV types were assigned to species using conventional taxonomic criteria [5,6]. Briefly, using the L1 ORF of papillomaviruses, viruses with <60% sequence homology in this region are considered to be in a different genus. Sequence homology between 60 and 70% constitutes different species, while different HPV types within a species have between 70 and 89% nucleotide identity [5,6]. Only HPV types in the alpha-papillomavirus genus were considered in our study.

Univariate logistic regression was used to determine association between CIN3 lesions (as opposed to CIN2) and three categories of HPV co-infection, separately. The HPV co-infection categories were: 1) two or more HPV types from two or more species, 2) two or more HPV types from a single species, 3) two or more HPV types from a single high-risk species (α5, α6, α7, α9 or α11 [3]).

We also tested the association between CIN3 lesions and variables previously associated with CIN progression; namely: baseline CD4 count, patient age at the time of screening, patient age at the time of first intercourse, and lifetime number of sexual partners [12]. Univariate logistic regression was performed between presence of CIN3 and each variable. Univariate analyses were used to separately model the three categories of patient HPV as the dependent variable and patient CD4 counts either at presentation or the last available CD4 value as the independent variables.

Multivariate logistic regression models of CIN3 were tested, separately using the 3 HPV co-infection categories above, along with other variables statistically significant in univariate analyses. Presence of HPV 16 or 18, or the presence of a high-risk HPV of any type was also included in the model.

### Study population

94 women with CIN2, CIN3, and squamous cell carcinoma made up the final study population. 41 of the patients had CIN2, 52 had CIN3, and one had squamous cell carcinoma (SCC). The squamous cell carcinoma patient was categorized as CIN3 in the logistic regression analyses (ordinal analyses with a separate SCC category yielded comparable results, Additional file 1: Table S1). The mean age of study participants was 36.78 and the median number of HPV type co-infections per patient was 3 (Table 1).

**Table 1:** Summary statistics of study population

**Variable**	**Mean (st dev)**	**Median (IQR)**
Age	36.78 (5.90)	36 (33-39)
HPV infections per person	3.93 (2.62)	3 (2-5)
Age at first intercourse*	18.13 (2.32)	18 (16-20)
Number of lifetime sexual partners*	5.86 (5.26)	4 (3-6)
CD4 count during first visit**	254.85 (207.29)	200 (109-365)
CD4 count during last visit	452.43 (208.12)	426 (285-578)

Data available for 63 patients* and for 61 patients**.

The classification of HPV types into species is presented in Additional file 1: Table S2 [13]. 79 patients were infected with multiple HPV types. 72 patients had two or more HPV types from different species, 53 patients had two or more HPV types from a single species, and 40 patients had two or more HPV types from a single high-risk species. We found co-infections with only single HPV types of multiple species in 26 patients. Seven patients were infected with multiple HPV types from one species exclusively, all of which were high-risk species. 46 patients had infections with both HPV types in multiple species and multiple HPV types in the same species. 90 patients were infected with a high-risk HPV type, while 50 patients had an infection with either HPV 16 or 18.

### Univariate analyses

Infection with two or more HPV types from a single species protected against progression to higher risk lesions (OR = 0.41, 95% CI 0.18-0.97, p = 0.042; Table 2). Infection with two or more HPV from a single high-risk species was also protective (OR = 0.37, 95% CI 0.16-0.86, p = 0.021). Infection with two or more HPV types from different species did not associate with progression (OR = 0.68, 95% CI 0.25-1.81, p = 0.435).

**Table 2:** Univariate and multivariate logistic regression results modeling CIN3 presence/absence or HPV co-infections

**Outcome**	**Covariate(s)**	**Odds ratio**	**95% CI**	**p value**
Presence of CIN3	Multiple types single species	0.4145	0.177-0.970	0.042
Presence of CIN3	Multiple types different species	0.6753	0.252-1.808	0.435
Presence of CIN3	Multiple types single high-risk species	0.3696	0.159-0860	0.021
Presence of CIN3	HPV 16 or 18	0.8127	0.358-1.843	0.620
Presence of CIN3	Age	1.0204	0.951-1.095	0.576
Presence of CIN3	Sexual Partners*	0.9997	0.909-1.099	0.994
Presence of CIN3	Age at First Sex*	1.0722	0.861-1.336	0.534
Presence of CIN3	CD4 count at presentationϮ	1.0014	0.998-1.004	0.297
Presence of CIN3	Last available CD4 count	1.0003	0.998-1.002	0.746
Presence of CIN3	Multiple types from single species; high-risk HPVǂ	0.3783	0.159-0.899	0.028
Presence of CIN3	Multiple types from single species; HPV 16 or 18	0.4138	0.172-0.996	0.049
Presence of CIN3	Multiple types from different species; high-risk HPVǂ	0.6061	0.225-1.632	0.322
Presence of CIN3	Multiple types from different species; HPV 16 or 18	0.6980	0.256-1.900	0.482
Presence of CIN3	Multiple types from single high-risk species; high-risk HPVǂ	0.4158	0.177-0.975	0.044
Presence of CIN3	Multiple types from single high-risk species; HPV 16 or 18	0.3661	0.155-0.847	0.024
2+ HPV infections	CD4 count at presentation	0.9978	0.995-1.001	0.168
2+ HPV infections	Last available CD4 count	0.9980	0.995-1.001	0.130
Multiple types single species	CD4 count at presentation	1.0001	0.998-1.002	0.963
Multiple types single species	Last available CD4 count	0.9995	0.998-1.001	0.618
Multiple types single high-risk species	CD4 count at presentation	1.0006	0.998-1.003	0.623
Multiple types single high-risk species	Last available CD4 count	1.0008	0.999-1.003	0.413
Multiple types different species	CD4 count at presentation	0.9980	0.995-1.001	0.187
Multiple types different species	Last available CD4 count	0.9981	0.996-1.000	0.106

Data available for 63 patients* and for 61 patientsϮ. 90 patients had high risk HPV typesǂ.

Univariate regression of CIN3 with a patient’s CD4 count at first presentation, last available CD4 count, patient’s age, number of lifetime sexual partners, and most of the individual high-risk HPV types were not statistically significant (Table 2, Additional file 1: Table S3). Patients with HPV types 39 were always diagnosed with CIN3, and those with HPV 26, 40, or 73 always had CIN2 (Additional file 1: Table S3). HPV 51 also associated with CIN3 (OR = 0.16, 95% CI 0.03-0.81, p = 0.027) (Additional file 1: Table S3). In our sample, infection with either HPV 16 or 18 was not associated with progression to CIN3 (p = 0.62). Initial and last available CD4 counts were not associated with the presence of any of the HPV co-infection categories (Table 2).

### Multivariate analyses

In multivariate analysis, the association of CIN3 with two or more HPV type infections from multiple species was not significant when adjusting for high-risk HPV type infections (OR = 0.61, 95% CI 0.22-1.63, p = 0.322; Table 2). Association of CIN3 with two or more HPV type infections from a single species was significant when adjusting for high-risk HPV type infections (single species co-infection OR = 0.38, 95% CI 0.16-0.90, p = 0.028; Table 2). Infections with two or more HPV types from a single high-risk species were significant in multivariate analyses adjusted for all high-risk HPV (OR = 0.42, 95% CI 0.18-0.97, p = 0.044; Table 2).

## Discussion

Our results indicate that the presence of infections with two or more HPV types from the same species protects from progression to high-grade CIN. While our data did not longitudinally ascertain the sequence of infection events, it is likely that some portion of our patients were infected with a low-risk HPV type prior to the acquisition of a high-risk type from the same species. This sequence of events could boost the immune system against the high-risk types and provide cross-protection via a mechanism similar to that afforded for HPV 31 and 45 infections when using vaccines [14].

Cross-reactive immune response based on taxonomic placement has been previously considered among HPV vaccines [15]. Immune responses to natural infection have been shown to be weaker than those elicited by the vaccines [14]. However, the persistence of HPV infections coupled with the immunodominant nature of the response, generating antibodies to only a subset of antigens, makes natural cross-protection an important factor to consider [16]. Cross-reactivity could offer a degree of protection capable of preventing or delaying carcinogenesis to only a subset of similar type infections (same species) but not strong enough to clear the infection. This mechanism could account for the effects observed in our study. This conclusion is further supported by the result that co-infections with HPV types from different species did not associate with protection for progressing to CIN3, while univariate analyses of same species infections in only the five high-risk species provided stronger associations and effect sizes than same species infections in general (Table 2, Additional file 1: Table S1). Hence sequence similarity among co-infections increases protection.

HIV and resultant low CD4 counts have been previously associated with increased risk of single and multiple HPV infections [17]. However, in our study, HIV-associated immunosuppression was not predictive of multiple HPV infections (Table 2). We observed no effect of CD4 counts on the likelihood of acquisition of co-infections with multiple HPV species, regardless of category (Table 2). This leads us to suggest that our findings are applicable beyond the niche of HIV + patients.

Our data, observed in an unvaccinated, natural setting, indicate that protection from any high-risk species type is likely to confer some degree of protection against all types in that species, supporting the argument that vaccines would do better if all high-risk species were covered. Therefore, widespread use of HPV vaccines can indirectly affect the prevalence of some non-vaccine types. Since the bivalent vaccine covers species α7 and α9, and the quadrivalent adds protection to α10, the burden of HPV carcinogenesis in a fully vaccinated population might shift to the high-risk species that are not currently covered by either vaccine; namely α5, α6, and α11. Adding virus-like particles from at least one HPV type in each of these species should therefore be a priority for future vaccine development.

This study was reviewed and approved by the Institutional Review Boards of the University of Pennsylvania and the Botswana Ministry of Health.

## Competing interests

The authors declare that they have no competing interests.

## Authors’ contributions

NZ and RS had full access to all the data in the study and take responsibility for the integrity of the data and the accuracy of the data analysis. Study concept and design: RS, DR, SW and NZ. Acquisition of data: NZ and DR. Analysis and interpretation of data: RS, DR, SW and NZ. Drafting of manuscript: RS. Critical revision of the manuscript for important intellectual content: RS, DR, SW and NZ. Statistical analysis: RS, SW and NZ. Obtaining funding: NZ. Administrative, technical, or material support: DR and NM. Study supervision: SW and NZ. All authors read and approved the final manuscript.

## Supplementary Material

Additional file 1: Table S1 Univariate and multivariate ordinal regression modeling lesion severity, CIN2 = 0, CIN3 = 1, and SCC = 2, with co-infection categories and relevant covariates. **Table S2.** Assignment of HPV types found in this study into species of the alpha-papillomavirus genus. **Table S3.** Univariate logistic regression results of progression to CIN 3 versus individual HPV types. Click here for file
