# Supplementary material for: Co-infection with HPV types from the same species provides natural cross-protection from progression to cervical cancer
Source: Infect Agent Cancer. 2014 Aug 12;9:26. doi: 10.1186/1750-9378-9-26 (PMC4141127; doi:10.1186/1750-9378-9-26)
Supplement: Additional file 1: Table S1 — Univariate and multivariate ordinal regression modeling lesion severity, CIN2 = 0, CIN3 = 1, and SCC = 2, with co-infection categories and relevant covariates. Table S2. Assignment of HPV types found in this study into species of the alpha-papillomavirus genus. Table S3. Univariate logistic regression results of progression to CIN 3 versus individual HPV types. [file 1750-9378-9-26-S1.doc]

**Table S1.** Univariate and multivariate ordinal regression modeling lesion severity, CIN2=0, CIN3=1, and SCC=2, with co-infection categories and relevant covariates

| Outcome | Covariate(s) | p value | Coefficient | 95%CI |
| --- | --- | --- | --- | --- |
| presence of CIN3 | Multiple types single species | 0.033 | -0.92 | - 1.76 - (-0.07) |
| presence of CIN3 | Multiple types different species | 0.324 | -0.5 | -1.49 - 0.49 |
| presence of CIN3 | Multiple types single high-risk species | 0.018 | -1.02 | -1.85 - (-0.18) |
| presence of CIN3 | Multiple types from single species; high-risk HPV | 0.022 | -1 | -1.86 - (-0.14) |
| presence of CIN3 | Multiple types from single species; HPV 16 or 18 | 0.041 | -0.91 | -1.78 - (-0.04) |
| presence of CIN3 | Multiple types from different species; high-risk HPV | 0.233 | -0.61 | -1.62 - 0.39 |
| presence of CIN3 | Multiple types from different species; HPV 16 or 18 | 0.368 | -0.46 | -1.47 - 0.55 |
| presence of CIN3 | Multiple types from single high-risk species; high-risk HPV | 0.036 | -0.91 | -1.76 - (-0.06) |
| presence of CIN3 | Multiple types from single high-risk species; HPV 16 or 18 | 0.022 | -1.01 | -1.88 - (-0.15) |

**Table S2.** Assignment of HPV types found in this study into species of the alpha-papillomavirus genus

| Species | HPV types |
| --- | --- |
| 1 | 42 |
| 3 | 61, 62, 72, 81, 83, 84 |
| 5 | 26, 51, 69, 82 |
| 6 | 53, 56, 66 |
| 7 | 18, 39, 45, 59, 68, 70 |
| 8 | 40 |
| 9 | 16, 31, 33, 35, 52, 58, 67 |
| 10 | 6, 11, 55 |
| 11 | 73 |
| 13 | 54 |
| 15 | 71 |

**Table S3.** Univariate logistic regression results of progression to CIN 3 versus individual HPV types

| Outcome | Covariate(s) | p value | Odds Ratio | 95%CI |
| --- | --- | --- | --- | --- |
| presence of CIN3 | HPV -6 | 0.256 | 0.3627 | 0.063-2.086 |
| presence of CIN3 | HPV -11 | 0.456 | 0.4967 | 0.079-3.121 |
| presence of CIN3 | HPV -16 | 0.429 | 1.4135 | 0.599-3.333 |
| presence of CIN3 | HPV -18 | 0.251 | 0.5579 | 0.206-1.510 |
| presence of CIN3 | HPV -26 | predicts CIN2 perfectly |  |  |
| presence of CIN3 | HPV -31 | 0.164 | 0.3965 | 0.108-1.461 |
| presence of CIN3 | HPV -33 | 0.194 | 0.4939 | 0.170-1.432 |
| presence of CIN3 | HPV -35 | 0.874 | 0.9314 | 0.386-2.248 |
| presence of CIN3 | HPV -39 | predicts CIN3 perfectly |  |  |
| presence of CIN3 | HPV -40 | predicts CIN2 perfectly |  |  |
| presence of CIN3 | HPV -42 | 0.458 | 0.5550 | 0.117-2.631 |
| presence of CIN3 | HPV -45 | 0.089 | 0.2914 | 0.070-1.207 |
| presence of CIN3 | HPV -51 | 0.027 | 0.1618 | 0.032-0.809 |
| presence of CIN3 | HPV -52 | 0.704 | 0.7551 | 0.177-3.220 |
| presence of CIN3 | HPV -53 | 0.634 | 0.7447 | 0.221-2.505 |
| presence of CIN3 | HPV -54 | 0.745 | 0.7600 | 0.145-3.977 |
| presence of CIN3 | HPV -55* | collinear |  |  |
| presence of CIN3 | HPV -56 | 0.144 | 0.2824 | 0.052-1.537 |
| presence of CIN3 | HPV -58 | 0.148 | 0.5051 | 0.200-1.275 |
| presence of CIN3 | HPV -59 | 0.256 | 0.3627 | 0.063-2.086 |
| presence of CIN3 | HPV -61 | 0.260 | 0.5511 | 0.196-1.553 |
| presence of CIN3 | HPV -62 | 0.991 | 0.9935 | 0.336-2.938 |
| presence of CIN3 | HPV -66 | 0.320 | 1.8920 | 0.539-6.646 |
| presence of CIN3 | HPV -67 | 0.854 | 0.7692 | 0.047-12.679 |
| presence of CIN3 | HPV -68 | 0.668 | 0.7500 | 0.202-2.787 |
| presence of CIN3 | HPV -69 | 0.854 | 0.7692 | 0.047-12.679 |
| presence of CIN3 | HPV -70 | 0.130 | 3.4667 | 0.695-17.304 |
| presence of CIN3 | HPV -71 | 0.800 | 0.8864 | 0.349-2.251 |
| presence of CIN3 | HPV -72 | 0.758 | 1.1932 | 0.387-3.673 |
| presence of CIN3 | HPV -73 | predicts CIN2 perfectly |  |  |
| presence of CIN3 | HPV -81 | 0.716 | 1.3194 | 0.296-5.874 |
| presence of CIN3 | HPV -82 | 0.081 | 0.2288 | 0.044-1.200 |
| presence of CIN3 | HPV -83 | 0.256 | 0.3627 | 0.063-2.086 |
| presence of CIN3 | HPV -84 | 0.704 | 0.7551 | 0.177-3.220 |

*11 observations
